# Molecular Detection of Two Potential Probiotic Lactobacilli Strains and Evaluation of Their Performance as Starter Adjuncts in Yogurt Production

**DOI:** 10.3390/ijms17050668

**Published:** 2016-05-04

**Authors:** Georgia Saxami, Olga S. Papadopoulou, Nikos Chorianopoulos, Yiannis Kourkoutas, Chrysoula C. Tassou, Alex Galanis

**Affiliations:** 1 Department of Molecular Biology and Genetics, Democritus University of Thrace, Alexandroupolis 68100, Greece; saxamigeorgia@hotmail.gr (G.S.); ikourkou@mbg.duth.gr (Y.K.); 2 Institute of Technology of Agricultural Products, Hellenic Agricultural Organization—DEMETER, Sof. Venizelou 1, Lycovrissi, Attiki 14123, Greece; olga_papadopoulou@aua.gr (O.S.P.); ctassou@nagref.gr (C.C.T.)

**Keywords:** probiotics, lactic acid bacteria, multiplex PCR, yogurt

## Abstract

A molecular method for efficient and accurate detection and identification of two potential probiotic lactobacilli strains isolated from fermented olives, namely *Lactobacillus pentosus* B281 and *Lb. plantarum* B282, was developed in the present study. Random Amplified Polymorphic DNA (RAPD) analysis was performed, and strain specific primers were designed and applied in a multiplex polymerase chain reaction (PCR) assay. The specificity of the assay was tested and successfully confirmed in 27 and 22 lactobacilli strains for *Lb. pentosus* B281 and *Lb. plantarum* B282, respectively. Moreover, the two strains were used as starter cultures in yogurt production. Cell enumeration followed by multiplex PCR analysis demonstrated that the two strains were present in yogurt samples at levels ≥6 log CFU/g even after 35 days of storage at 4 °C. Microbiological analysis showed that lactobacilli and streptococci were present within usual levels, whereas enterobacteriaceae and yeast/mold counts were not detected as expected. Although the pH values of the novel products were slightly lower than the control ones, the yogurt containing the probiotic cultures scored similar values compared to the control in a series of sensory tests. Overall, these results demonstrated the possible use of the two strains as starter adjuncts in the production of yogurt with potential probiotic properties.

## 1. Introduction

Nowadays the theory that regular consumption of probiotic food products is linked to enhanced health and longevity is well established. The relief of symptoms caused by several gastrointestinal disorders [1], the alleviation of lactose intolerance [2], the prevention of allergies [3], obesity [4,5], and osteoporosis [6], and the reduction of the risk of colon [7], breast [8], or bladder cancer [9] are among the beneficial health effects described in humans and animals. Accordingly, yogurt, cheese, ice cream, and other dairy products are commercially available [10]. At the same time, the food industry and the consumers show significant interest in the development of non-dairy probiotic products [11]. Indeed, the effective incorporation of probiotic microorganisms has been demonstrated in fruit juices [12], chestnuts [13], and dry fermented sausages [14].

In addition, a recent study showed that several lactic acid bacteria (LAB) strains isolated from naturally fermented table olives display probiotic potential [15]. Among them, two strains, namely *Lactobacillus pentosus* B281 and *Lactobacillus plantarum* B282, were used effectively as starter cultures in Spanish-style green olive fermentation [16,17]. Significantly, both strains colonized the olive surface at populations ranging from 10^6^ to 10^7^ CFU/g throughout fermentation, without affecting the physicochemical properties of the product. Moreover, the novel probiotic olives were accepted for consumption, as indicated by the sensory evaluation tests performed [16]. However, further studies are required to confirm and establish the probiotic character of the two strains.

Monitoring the presence of LAB in food products is crucial in order to evaluate their probiotic character and elucidate their mechanisms of action. To this end, several polymerase chain reaction (PCR)-based molecular methods have been presented with the power to detect, identify, and distinguish the microorganisms of interest among closely related species and strains [18]. For example, multiplex PCR assays have been developed for accurate and efficient detection of specific LAB strains in probiotic products [19,20,21]. The design of strain specific primers was performed by comparative sequence analysis. However, when sequence information is not available the Random Amplified Polymorphic DNA (RAPD) technique may be firstly employed as a suitable means by which to reveal the needed polymorphism [22,23]. Short arbitrary primers (a 10-base pair sequence) are used to generate multiple randomly sized DNA fragments in PCR reactions with low-stringency annealing conditions. The sequence-characterized amplified regions (SCAR) produced are then used to design strain-specific PCR primers. Of note, we have recently applied this methodology for the molecular detection of two specific LAB strains with potential probiotic properties [24].

The aim of this study was the design and development of a multiplex PCR assay based on RAPD analysis for the detection of strains *Lb. pentosus* B281 and *Lb. plantarum* B282 in a single reaction. The accuracy and efficiency of the assay were tested against several different LAB strains. Moreover, the developed methodology was applied to monitoring the presence of the two strains during refrigerated storage of yogurt prepared with the two strains as starter cultures. Finally, the microbiological, physicochemical, and sensory properties of the products were studied, to evaluate the performance of the two strains as starter cultures for the production of novel probiotic dairy products.

## 2. Results and Discussion

### 2.1. Screening of Random Amplified Polymorphic DNA (RAPD) Primers, Isolation of Sequence-Characterized Amplified Regions (SCAR) Markers, and Design of Novel Primers for Multiplex Polymerase Chain Reaction (PCR)

**Figure 1:**
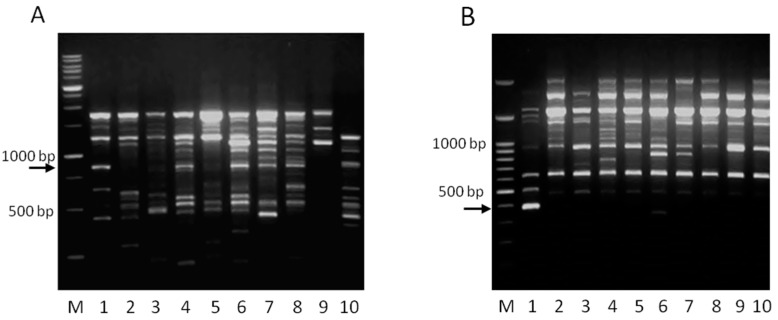
Random Amplified Polymorphic DNA (RAPD) analysis for *Lb. pentosus* B281 and *Lb. plantarum* B282. (**Α**) Electrophoretic profile of *Lb. pentosus* B281 generated with RAPD primer AG281 (lane 1), and 9 *Lb. pentosus* strains (lanes 2–10). Lanes: 2, *Lb. pentosus* E95; 3, *Lb. pentosus* E106B; 4, *Lb. pentosus* E128; 5, *Lb. pentosus* E89; 6 *Lb. pentosus* E119; 7, *Lb. pentosus* E182; 8, *Lb. pentosus* E105; 9, *Lb. pentosus* 632; 10, *Lb. pentosus* 612. M: 1 kb DNA ladder. The numbers on the left of the figure indicate the DNA size markers in base pairs (bp). The arrow indicates the band of 872 bp; (**Β**) Electrophoretic profile of *Lb. plantarum* B282 generated with RAPD primer AG282 (lane 1), and 9 *Lb. plantarum* strains (lanes 2–10). Lanes: 2, *Lb. plantarum* E4; 3, *Lb. plantarum* E1; 4, *Lb. plantarum* E45; 5, *Lb. plantarum* E50; 6, *Lb. plantarum* E66; 7, *Lb. plantarum* E68; 8, *Lb. plantarum* E71; 9, *Lb. plantarum* E73; 10, *Lb. plantarum* E77. M: 100 bp DNA ladder. The numbers on the left of the figure indicate the DNA size markers in base pairs (bp). The arrow indicates the band of 391 bp.

A total of 123 arbitrary primers were tested with RAPD PCR with DNA extracted from pure cultures of *Lb. pentosus* B281 and *Lb. plantarum* B282. Thirty-two primers for *Lb. pentosus* B281 and 24 primers for *Lb. plantarum* B282 produced more than five scorable bands and were selected for further analysis. Out of the primers selected, primers AG281 and AG282 gave unique RAPD profiles for the two strains, as presented in Figure 1. To confirm the reproducibility of the method, each reaction was repeated three times with the same conditions. Two potential strain specific RAPD markers, an 872 base pairs (bp) band for *Lb. pentosus* B281 (Figure 1A) and a 391 bp band for *Lb. plantarum* B282 (Figure 1B), were isolated from the agarose gel, cloned into an appropriate pBlueScript vector, and sequenced.

Potential strain-specific primers for *Lb. pentosus* B281 and *Lb. plantarum* B282 were designed with the obtained nucleotide sequences. The nucleotide sequences for the primers of *Lb. pentosus* B281 were 5′-GGTGAAGCTGATATTTATG-3′ for 281F and 5′-GGTGAAGCTGGTGGTGGTATC-3′ for 281R. In addition, the nucleotide sequences for the primers of *Lb. plantarum* B282 were 5′-CCACAGCAGTAGGGCGCGAG-3′ for 282F and 5′-CCACAGCAGTCTGCCCAACC-3′ for 282R. A multiplex PCR assay was developed employing the novel primer pair for each strain of interest and a set of primers (P1 and P2) that recognizes the *16S rRNA* gene of LAB, and gives an 89 bp product that serves as a positive control marker [25]. For reaction optimization, the step-by-step protocol proposed by Henegariu *et al.* [26] was followed.

### 2.2. Specificity of the Multiplex PCR

**Figure 2:**
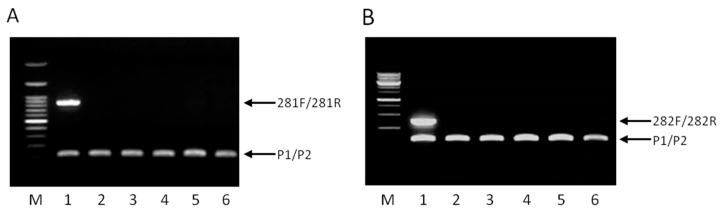
Agarose gel electrophoresis of PCR products from multiplex PCR assay. (**A**) Lanes: 1, *Lb. pentosus* B281; 2, *Lb. pentosus* E95; 3, *Lb. pentosus* E106B; 4, *Lb. pentosus* E128; 5, *Lb. pentosus* E89. M: 100 bp DNA ladder. The PCR product generated from the primer set 281F/281R of 872 bp is unique to *Lb. pentosus* B281, whereas the PCR product of the primer set P1/P2 of 89 bp is universal for lactobacilli. Both products are indicated with the corresponding arrows; (**B**) Lanes: 1, *Lb. plantarum* B282; 2, *Lb. plantarum* E4; 3, *Lb. plantarum* E1; 4, *Lb. plantarum* E45; 5, *Lb. plantarum* E50; 6, *Lb. plantarum* E66. M: 1 kb DNA ladder. The PCR product generated from the primer set 282F/282R of 391 bp is unique to *Lb. plantarum* B282, whereas the PCR product of the primer set P1/P2 of 89 bp is universal for lactobacilli. Both products are indicated with the corresponding arrows.

The multiplex PCR was initially tested with DNA extracted from *Lb. pentosus* B281 and five other *Lb. pentosus* strains also isolated from table olives [27]. As presented in Figure 2A, *Lb. pentosus* B281 gave two distinct products (89 and 872 bp), whereas only one, the 89 bp positive control product, was produced for the five other *Lb. pentosus* strains tested (Figure 2A). Similarly, a multiplex PCR with the novel primers 282R and 282F and the universal primers P1 and P2 was performed with DNA from *Lb. plantarum* B282 and five other *Lb. plantarum* strains isolated from table olives. Significantly, two PCR products were only produced (391 and 89 bp) for *Lb. plantarum* B282, enhancing the specificity of the reaction (Figure 2B).

To further validate the specificity of our methodology, multiplex PCR assays employing the RAPD-derived primers for *Lb. pentosus* B281 and the universal primers P1 and P2 were performed with DNA extracted from *Lb. pentosus* B281 and 22 *Lb. pentosus* wild-type strains isolated from table olives, as well as from five other LAB strains, including the reference strains *Lb. casei* Shirota ACA-DC 6002, *Lb. rhamnosus* GG ATCC 53103, and *Lb. casei* ATCC 393, which are commonly used in probiotic products. A two-band pattern was exclusively generated for *Lb. pentosus* B281 (Table S1). In parallel, multiplex PCR reactions were performed on *Lb. plantarum* B282 and 22 different LAB strains, including eight *Lb. plantarum* and five *Lb. pentosus* wild-type strains isolated from table olives, using the novel primers for *Lb. plantarum* B282 and the universal primers P1 and P2. As expected, two products were generated only for *Lb. plantarum* B282 (Table S2). To determine the detection limit of our proposed methodology, DNA from pure cultures containing the strain of interest at levels ≥10^5^, ≥10^4^, and ≥10^3^ cells/mL, respectively, was extracted and multiplex PCR reactions were performed. Both *Lb. pentosus* B281 and *Lb. plantarum* B282 were detected at levels of ≥10^4^ cells/mL (data not shown).

### 2.3. Μicrobiological Analysis

**Figure 3:**
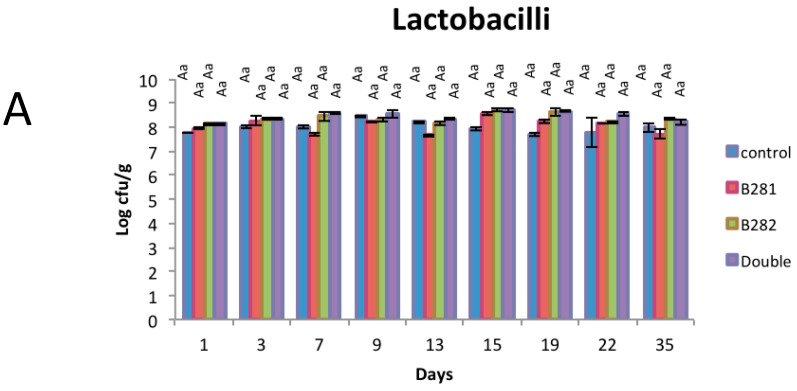

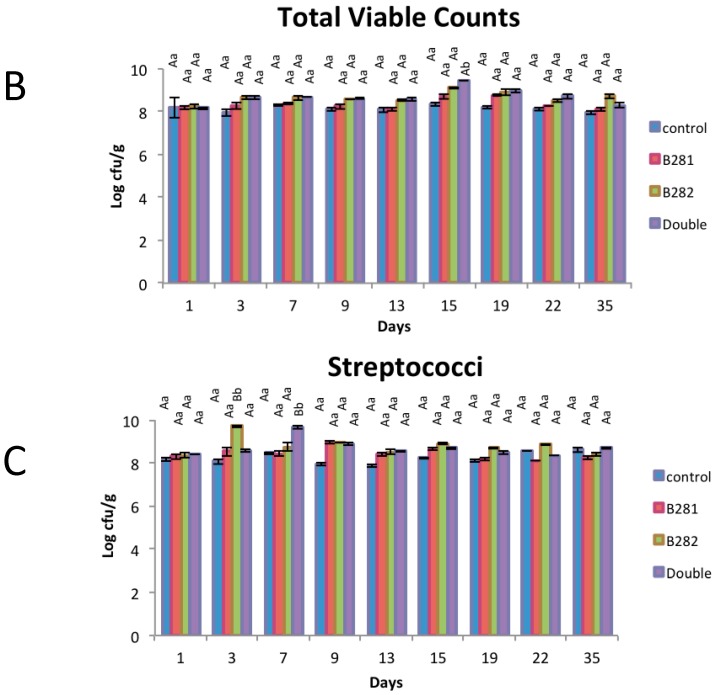
(**A**) Lactobacilli; (**B**) total viable counts; and (**C**) streptococci in yogurt samples during refrigerated storage. Means with different uppercase letters within the same treatment are significantly different (*p* < 0.05). Means with different lowercase letters within the same storage day are significantly different (*p* < 0.05).

*Lb. pentosus* B281 and *Lb. plantarum* B282 alone or in combination were employed as starter cultures in yogurt production and microbiological analysis was performed during the storage of the products. Lactobacilli, streptococci, and total viable counts in the yogurt samples are presented in Figure 3. Numbers of lactobacilli, for probiotic cases, increased during the first days of storage and then the behavior of lactobacilli was similar until the end of shelf-life of the products. For the control case, the lactobacilli population (starter culture) was found at lower levels than those of probiotics for the first two sampling days, while it decreased until the end of shelf-life of yogurt. Streptococci levels remained almost stable during refrigerated storage (approximately 8 log CFU/g) for all cases. Enterobacteriaceae and yeast/mold counts were not detected in any yogurt sample during the whole period of the experiment (data not show). These microbiological results are in accordance with those of a previous study from our group using free or immobilized cells on whey protein of a different probiotic LAB strain, as co-culture during the pilot production of either bovine or ewe’s milk yogurt in industry [28].

### 2.4. Monitoring the Presence of Lb. pentosus B281 and Lb. plantarum B282 in Yogurt

It is generally considered that the minimum number of probiotic cells in food products required to provide a healthy benefit is ≈6 log CFU/g [29,30]. Therefore, we investigated whether *Lb. pentosus* B281 and *Lb. plantarum* B282 were present in yogurt samples above this threshold level during refrigerated storage. After lactobacilli enumeration, the presence of the two strains in petri dishes corresponding to the concentration of ≥6 log CFU/g was detected by multiplex PCR using the novel strain-specific primers. It was found that both strains were present at these levels in the corresponding yogurt samples after 1, 15, and 35 days of storage at 4 °C (Table 1). The high survival rates of probiotic cultures observed in the present study could be attributed to the acid-resistant nature of the strains used. Similar results regarding the survival of probiotic cultures in yogurt after refrigerated storage for long time periods have been observed in other studies too [31,32,33], although a significant decline in viable counts was observed in many cases, resulting in levels below 6 log CFU/g [34,35].

### 2.5. pH Determination

**Figure 4:**
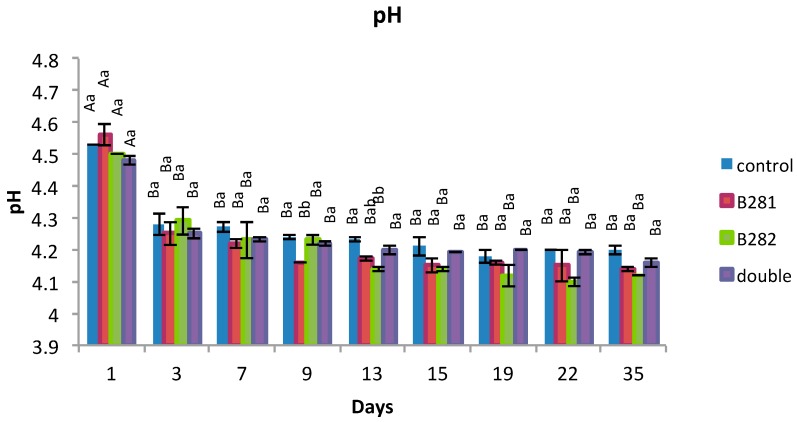
pH Values in yogurt samples during refrigerated storage. Means with different uppercase letters within the same treatment are significantly different (*p* < 0.05). Means with different lowercase letters within the same storage day are significantly different (*p* < 0.05).

The results of pH determination are presented in Figure 4. pH values ranged at usual levels and were significantly (*p* < 0.05) affected by all factors (probiotic culture and storage time). It is evident that during storage pH values declined in all cases, but yogurts with probiotic cultures were found to be more acidic in comparison with the controls.

### 2.6. Sensory Evaluation

**Figure 5:**
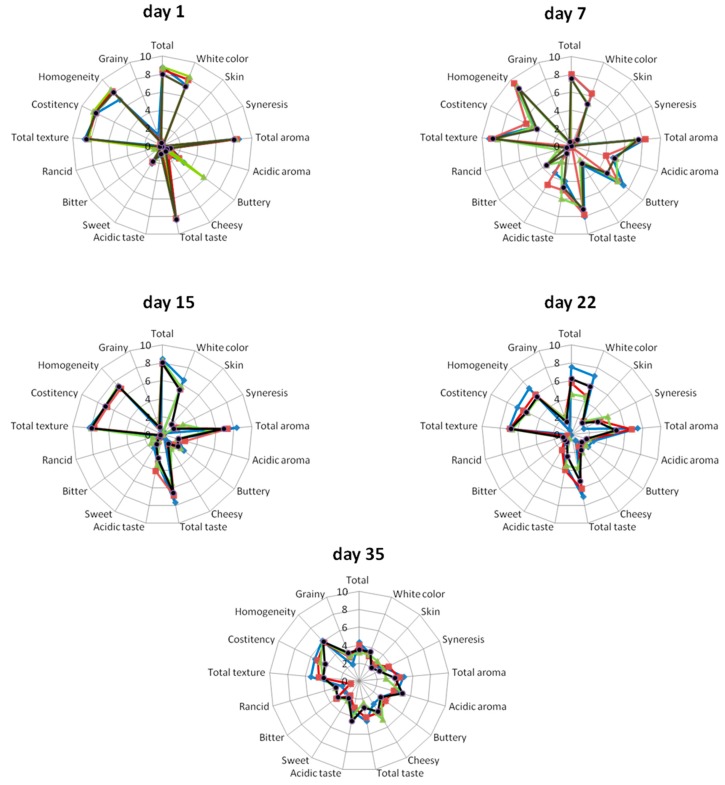
Sensory evaluation of yogurts during refrigerated storage at 4 °C (sampling day 1, 7, 15, 22, and 35). (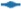): Yogurt produced with no probiotic culture; (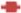): Yogurt containing *Lb. pentosus* B281; (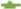): Yogurt containing *Lb. plantarum* B282; (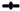): Yogurt containing *Lb. pentosus* B281 and *Lb. plantarum* B282.

The results of the organoleptic assessment for sampling days 1, 7, 15, 22, and 35 were presented in Figure 5. In general, the panelists attributed similar total evaluation scores (*p* > 0.05) to the new probiotic and the control products, during refrigerated storage (Figure 5). The supplementation of yogurt with probiotic cultures of *Lb. pentosus* B281 and both *Lb. pentosus* B281 and *Lb. plantarum* B282 led to similar scores for total taste and total appearance of the products and the control samples, during two weeks of cold storage. In this respect, the 10-member taste panel found that the organoleptic characteristics of probiotic yogurt for all cases on the 22nd day of storage were still evaluated positively (Figure 5) and were found to be more acidic compared to the controls. Yogurt containing B281 strain was found sweeter at the 7th and 22nd day compared to the control case and the other probiotic cases. On the other hand, all yogurts were evaluated negatively at the 35th day of storage. Concerning total texture and rancidity, probiotic yogurts had similar values to the control products (*p* > 0.05). Yogurts with the addition of *Lb. pentosus* B281 had similar values (*p* > 0.05) for homogeneity, total taste, total aroma, and total appearance to the controls after two weeks of cold storage (Figure 5).

## 3. Experimental Section

### 3.1. Bacterial Strains and Culture Conditions

All *Lb. pentosus* and *Lb. plantarum* strains, were isolated previously from industrially fermented olives [27]. Cultures of *Lb. pentosus* B281 and *Lb. plantarum* B282 were activated from a stock culture stored at −80 °C in MRS broth and were grown at 30 °C for 24 h in de Man, Rogosa, and Sharpe (MRS) broth (Fluka, Buchs, Switzerland). A starter culture of *Streptococcus thermophilus* was grown at 37 °C for 24 h on M17 broth (Oxoid Limited, Hampshire, UK) and the strain *Lb. delbrueckii* ssp. bulgaricus was grown at 30 °C for 24 h anaerobically in MRS broth (Fluka). *Lb. paracasei* DSM 20207, *Lb. paracasei* DSM 46331 and *Lb. paracasei* DSM 5622 strains were grown at 30 °C in MRS broth (Fluka).

### 3.2. RAPD PCR

The RAPD primer generator application was employed for the design of the decamer RAPD primers [36]. All primers were obtained from VBC-Biotech, Vienna, Austria. PCR reactions were performed as described previously [24]. The PCR products were subjected to electrophoresis in 1.5% *w*/*v* agarose gels in Tris-acetate-EDTA (TAE) buffer. Staining of the gels was performed with 0.5 μg·mL^−1^ ethidium bromide. Then the gels were visualized under UV illumination and photographed with a digital camera (Gel Doc EQ System, Biorad, Segrate, Italy). Each reaction was repeated three times, and those producing reproducible fingerprints were considered for further marker purification and cloning.

### 3.3. Cloning and Sequencing

After electrophoresis separation of the RAPD fragments, the selected bands were excised from the gel, and DNA was isolated using a NucleoSpin Extract II kit (MACHEREY-NAGEL, Düren, Germany) according to the manufacturer’s instructions. The selected RAPD markers were cloned into the pBlueScript SK+ vector following the TA cloning protocol described by Zhou and Gomez-Sanchez [37], with slight modifications as described before [24]. Sequencing of the resulting clones was performed by VBC-Biotech, Austria. The nucleotide sequence of the cloned fragment was determined from three clones. With the obtained sequence, the forward and reverse primers were designed including the decamer primer sequence extending few nucleotides (8–10 bases) as described before [24].

### 3.4. Multiplex PCR

Multiplex PCR reactions were carried out as described before [24], with the employment of primers 281F: 5′-GGTGAAGCTGATATTTATG-3′ (10 pmol), and 281R: 5′-GGTGAAGCTGGTGGTGGTATC-3′ (10 pmol) for *Lb. pentosus* B281 and 282F: 5′-CCACAGCAGTAGGGCGCGAG-3′ (10 pmol) and 282R: 5′-CCACAGCAGTCTGCCCAACC-3′ (10 pmol), for *Lb. plantarum* B282, respectively. A set of primers consisted of P1: 5′-AGCAGTAGGGAATCTTCCA-3′ (10 pmol) and P2: 5′-ATTYCACCGCTACACATG-3′ (10 pmol) was also added as positive control [25]. Amplification was carried out in a Thermal Cycler (Eppendorf, Hamburg, Germany) under the following conditions: for *Lb. pentosus* B281, 95 °C (2 min), followed by 26 cycles of 95 °C (30 s), 63 °C (30 s), 72 °C (45 s), followed by a final extension step at 72 °C (2 min); for *Lb. plantarum* B282, 95 °C (2 min), followed by 22 cycles of 95 °C (30 s), 68 °C (30 s), 72 °C (45 s), followed by a final extension step at 72 °C (2 min). The PCR products were separated on 1% (*w*/*v*) agarose gels, visualized under UV illumination, and photographed with a digital camera (Gel Doc EQ System, BioRad).

### 3.5. Yogurt Production

Yogurts (100 g) were produced by inoculating pasteurized bovine milk with a typical yogurt culture consisting of *Lb. delbrueckii* ssp. *bulgaricus* and *Strep. thermophilus* (control), with *Lb. delbrueckii* ssp. *bulgaricus*, *Strep. thermophilus* and *Lb. pentosus* B281 (B281), *Lb. delbrueckii* ssp. *bulgaricus*, *Strep. thermophilus* and *Lb. plantarum* B282 (B282) and *Lb. delbrueckii* ssp. *bulgaricus*, *Strep. thermophilus*, *Lb. pentosus* B281, and *Lb. plantarum* B282 (double) and incubated in appropriate conditions (42 °C, 6 h). The initial cell counts for all cultures (*Lb. delbrueckii* ssp. *bulgaricus*, *Strep. thermophilus*, *Lb. pentosus* B281, and *Lb. plantarum* B282) were ~9 log CFU/g. After the fermentation process, samples were stored at 4 °C until the end of the shelf life of the product (*ca.* 35 days). Samples from each treatment were collected at various time intervals and subjected to microbiological, molecular, physicochemical, and sensory analyses.

### 3.6. Microbiological Analysis

Ten grams of duplicate yogurt samples were aseptically transferred to 90 mL sterilized quarter-strength Ringers solution (Sigma-Aldrich, Gillingham, UK). Serial dilutions were prepared with the Ringers solution and duplicate samples were spread or mixed on the following media: de Man–Rogosa–Sharp agar (MRS, CM 361, Oxoid, Oxford, UK) for LAB and incubated at 30 °C for 48 h; rose bengal chloramphenicol agar base (RBC, Biolife, Milano, Italy) for yeasts/molds incubated at 25 °C for 48–72 h; Violet Red Bile Glucose agar (Biolife) for *Enterobacteriaceae* overlaid with the same medium and incubated at 37 °C for 24 h; M17 agar (Biokar Diagnostics, Paris, France) for *Streptococcus thermophilus* and incubated at 37 °C for 48 h; plate count agar (Fluka) for total viable counts and incubated at 30 °C for 48 h.

### 3.7. Molecular Detection of Lb. pentosus B281 and Lb. plantarum B282 in Yogurt

Following enumeration of lactobacilli in MRS agar, the plates corresponding to the concentration of ≥6 log CFU/g (dilution factor 10^−5^) were washed with 1 mL sterilized quarter-strength Ringers solution (Sigma-Aldrich), and then the cell suspensions were subjected to molecular analysis based on multiplex PCR as described in Section 3.5. Genomic DNA from the lactobacilli suspensions was extracted using a DNeasy Tissue Kit (Qiagen, Hilden, Germany) according to the manufacturer’s protocol. The amount of extracted DNA was determined by absorbance at 260 nm using a UV spectrophotometer (Eppendorf).

### 3.8. pH Determination

In parallel with microbiological analysis the pH value of yogurt was evaluated using a pH-330i pH meter, WTW GmbH, Weilheim, Germany pH meter. The pH was recorded after the end of microbiological analysis by immersing the glass electrode in the yogurt sample.

### 3.9. Sensory Evaluation

Sensory evaluation of yogurt samples was performed during storage at the same time intervals as for microbiological analysis. Samples of approximately 25 g were served in a random order at room temperature. Sensory evaluation was performed by a panel of 10 members (trained staff from the Institute of Technology of Agricultural Products of Hellenic Agricultural Organization—DEMETER, Lycovrissi, Greece) after each sample, using locally approved protocols. The panel was asked to give scores on a 0 to 10 hedonic scale for attributes grouped into five categories: appearance, aroma, taste, texture, and overall quality. Assessment was designed to identify specific indicators (skin, white color, syneresis, acidic aroma, buttery, cheesy, acidic taste, sweet, bitter, rancid, consistency, homogeneity, and grainy) for each sensorial characteristic. Panelists used water to cleanse their palates between samples and were unaware of the identity of the samples they tasted. Sensory evaluation was carried out under controlled conditions of light, temperature, and humidity. Overall, two yogurt samples were scored for each replicate by the taste panel.

### 3.10. Statistical Analysis

All experiments were carried out in duplicate with two independent batches of yogurt each. Significance was established at *p* < 0.05. Results were analyzed for statistical significance with analysis of variance (ANOVA). Duncan’s multiple range test was used to determine significant differences among results (coefficients, ANOVA tables and significance (*p* < 0.05) were computed using Statistica v.6.0 Package, Statsoft, OK, USA).

## 4. Conclusions

In the present study a multiplex PCR assay was developed for detection and identification of two potential probiotic lactobacilli strains, namely *Lb. pentosus* B281 and *Lb. plantarum* B282, in a single reaction. This represents an efficient tool for accurate and rapid detection of the strains of interest in food products. The specific stains were originally isolated from olive microbiota and their performance as starters in the fermentation of green olives was evaluated previously. Here, their possible use in yogurt production was examined. Cell enumeration and employment of the novel multiplex PCR methodology showed that both strains were presented in yogurt samples at levels ≥6 log CFU/g during fermentation and storage. In addition, the microbiological, physicochemical, and sensory profiles of the novel products were similar to the controls. Although further *in vitro* and *in situ* studies are required, these results promote the use of the two strains as starter cultures for the production of novel dairy products with probiotic potential.

## Figures and Tables

**Table 1:** Detection of *Lb. pentosus* B281 and *Lb. plantarum* B282 in yogurt samples at levels ≥6 log CFU/g, after 1, 15, and 35 days of storage at 4 °C. Detection was performed by cell enumeration and multiplex polymerase chain reaction (PCR) analysis.

Yogurt Sample	Day 1		Day 15		Day 35	
	*Lb. pentosus* B281	*Lb. plantarum* B282	*Lb. pentosus* B281	*Lb. plantarum* B282	*Lb. pentosus* B281	*Lb. plantarum* B282
Control	−	−	−	−	−	−
B281	+	−	+	−	+	−
B282	−	+	−	+	−	+
Double	+	+	+	+	+	+

## Supplementary Materials

Supplementary materials can be found at http://www.mdpi.com/1422-0067/17/5/668/s1.

## References

[1] Varankovich N.V., Nickerson M.T., Korber D.R. (2015). Probiotic-based strategies for therapeutic and prophylactic use against multiple gastrointestinal diseases. Front. Microbiol..

[2] Li J., Zhang W., Wang C., Yu Q., Dai R., Pei X. (2012). *Lactococcus lactis* expressing food-grade β-galactosidase alleviates lactose intolerance symptoms in post-weaning Balb/c mice. Appl. Microbiol. Biotechnol..

[3] Cuello-Garcia C.A., Brożek J.L., Fiocchi A., Pawankar R., Yepes-Nuñez J.J., Terracciano L., Gandhi S., Agarwal A., Zhang Y., Schünemann H.J. (2015). Probiotics for the prevention of allergy: A systematic review and meta-analysis of randomized controlled trials. J. Allergy Clin. Immunol..

[4] Park D.Y., Ahn Y.T., Park S.H., Huh C.S., Yoo S.R., Yu R., Sung M.K., McGregor R., Choi M.S. (2013). Supplementation of *Lactobacillus curvatus* HY7601 and *Lactobacillus plantarum* KY1032 in diet-induced obese mice is associated with gut microbial changes and reduction in obesity. PLoS ONE.

[5] Poutahidis T., Kleinewietfeld M., Smillie C., Levkovich Τ., Perrotta Α., Bhela S., Varian B.J., Ibrahim Y.M., Lakritz J.R., Kearney S.M. (2013). Microbial reprogramming inhibits western diet-associated obesity. PLoS ONE.

[6] Britton R.A., Irwin R., Quach D., Schaefer L., Zhang J., Lee T., Parameswaran N., McCabe L.R. (2014). Probiotic *L. reuteri* treatment prevents bone loss in a menopausal ovariectomized mouse model. J. Cell. Physiol..

[7] Uccello M., Malaguarnera G., Basile F., D’agata V., Malaguarnera M., Bertino G., Vacante M., Drago F., Biondi A. (2012). Potential role of probiotics on colorectal cancer prevention. BMC Surg..

[8] Kaga C., Takagi A., Kano M., Kado S., Kato I., Sakai M., Miyazaki K., Nanno M., Ishikawa F., Ohashi Y. (2013). *Lactobacillus casei* Shirota enhances the preventive efficacy of soymilk in chemically induced breast cancer. Cancer Sci..

[9] Naito S., Koga H., Yamaguchi A., Fujimoto N., Hasui Y., Kuramoto H., Iguchi A., Kinukawa N. (2008). Prevention of recurrence with epirubicin and *lactobacillus casei* after transurethral resection of bladder cancer. J. Urol..

[10] Granato D., Branco G., Gomes Cruz A., de Assis Fonseca Faria J., Shah N. (2010). Probiotic dairy products as functional foods. Compr. Rev. Food Sci. Food Saf..

[11] Rivera-Espinoza Y., Gallardo-Navarro Y. (2010). Non-dairy probiotic products. Food Microbiol..

[12] Céspedes M., Cárdenas P., Staffolani M., Ciappini M.C., Vinderola G. (2013). Performance in nondairy drinks of probiotic *L. casei* strains usually employed in dairy products. J. Food Sci..

[13] Blaiotta G., di Capua M., Coppola R., Aponte M. (2012). Production of fermented chestnut purees by lactic acid bacteria. Int. J. Food Microbiol..

[14] Sidira M., Karapetsas A., Galanis A., Kanellaki M., Kourkoutas Y. (2014). Effective survival of immobilized *Lactobacillus casei* during ripening and heat treatment of probiotic dry-fermented sausages and investigation of the microbial dynamics. Meat Sci..

[15] Argyri A., Zoumpopoulou G., Karatzas K.A., Tsakalidou E., Nychas G.J., Panagou E., Tassou C. (2013). Selection of potential probiotic lactic acid bacteria from fermented olives by *in vitro* tests. Food Microbiol..

[16] Argyri A., Nisiotou A., Malouchos A., Panagou E., Tassou C. (2014). Performance of two potential probiotic *Lactobacillus* strains from the olive microbiota as starters in the fermentation of heat shocked green olives. Int. J. Food Microbiol..

[17] Blana V., Grounta A., Tassou C., Nychas G.J., Panagou E. (2014). Inoculated fermentation of green olives with potential probiotic *Lactobacillus pentosus* and *Lactobacillus plantarum* starter cultures isolated from industrially fermented olives. Food Microbiol..

[18] Mohania D., Nagpal R., Kumar M., Bhardwaj A., Yadav M., Jain S., Marotta F., Singh V., Parkash O., Yadav H. (2008). Molecular approaches for identification and characterization of lactic acid bacteria. J. Dig. Dis..

[19] Karapetsas A., Vavoulidis E., Galanis A., Sandaltzopoulos R., Kourkoutas Y. (2010). Rapid Detection and Identification of Probiotic *Lactobacillus casei* ATCC 393 by Multiplex PCR. J. Mol. Microbiol. Biotechnol..

[20] Nikolaou A., Saxami G., Kourkoutas Y., Galanis A. (2011). A new methodology for rapid detection of *Lactobacillus delbrueckii* subsp. bulgaricus based on multiplex PCR. J. Microbiol. Meth..

[21] Cremonesi P., Vanoni L., Morandi S., Silvetti T., Castiglioni B., Brasca M. (2011). Development of a pentaplex PCR assay for the simultaneous detection of *Streptococcus thermophilus*, *Lactobacillus delbrueckii* subsp. *bulgaricus*, *L. delbrueckii* subsp. *lactis*, *L. helveticus*, *L. fermentum* in whey starter for Grana Padano cheese. Int. J. Food Microbiol..

[22] Tilsala-Timisjärvi A., Tapani Alatossava T. (1998). Strain-specific identification of probiotic *Lactobacillus rhamnosus* with randomly amplified polymorphic DNA-derived PCR primers. Appl. Environ. Microbiol..

[23] Maruo T., Sakamoto M., Toda T., Benno Y. (2006). Monitoring the cell number of *Lactococcus lactis* subsp. *cremoris* FC in human feces by real-time PCR with strain-specific primers designed using the RAPD technique. Int. J. Food Microbiol..

[24] Galanis A., Kourkoutas Y., Tassou C.C., Chorianopoulos N. (2015). Detection and identification of probiotic *Lactobacillus plantarum* strains by multiplex PCR using RAPD-derived primers. Int. J. Mol. Sci..

[25] Klijn N., Weerkamp A.H., de Vos W.M. (1991). Identification of mesophilic lactic acid bacteria by using polymerase chain reaction-amplified variable regions of 16S rRNA and specific DNA probes. Appl. Environ. Microbiol..

[26] Henegariu O., Heerema N.A., Dlouhy S.R., Vance G.H., Vogt P.H. (1997). Multiplex PCR: Critical parameters and step-by-step protocol. Biotechniques.

[27] Doulgeraki A., Pramateftaki P., Argyri A., Nychas G.J., Tassou C., Panagou E. (2013). Molecular characterization of lactic acid bacteria isolated from industrially fermented Greek table olives. LWT Food Sci. Technol..

[28] Sidira M., Kiourtzidis M., Argyri A., Papadopoulou O., Chorianopoulos N., Tassou C., Kaloutsas S., Galanis A., Kourkoutas Y. (2016). Evaluation of immobilized *Lactobacillus plantarum* 2035 on whey protein as adjunct probiotic culture in yoghurt production. LWT Food Sci. Technol..

[29] Boylston T.D., Vinderola C.G., Ghoddusi H.B., Reinheimer J.A. (2004). Incorporation of bifidobacteria into cheeses: Challenges and rewards. Int. Dairy J..

[30] Oliveira M., Sodini I., Remeuf R., Tissier J.P., Corrieu G. (2002). Manufacture of fermented lactic beverages containing probiotic cultures. J. Food Sci..

[31] Donkor O.N., Henriksson A., Vasiljevic T., Shah N.P. (2007). Rheological properties and sensory characteristics of set-type soy yogurt. J. Agric. Food Chem..

[32] Sidira M., Saxami G., Dimitrellou D., Santarmaki V., Galanis A., Kourkoutas Y. (2013). Monitoring survival of *Lactobacillus casei* ATCC 393 in probiotic yogurts using an efficient molecular tool. J. Dairy Sci..

[33] Maragkoudakis P.A., Zoumpopoulou G., Miaris C., Kalantzopoulos G., Pot B., Tsakalidou E. (2006). Probiotic potential of *Lactobacillus* strains isolated from dairy products. Int. Dairy J..

[34] Vinderola C.G., Prosello W., Ghiberto T.D., Reinheimer J.A. (2000). Viability of probiotic (*Bifidobacterium*, *Lactobacillus acidophilus* and *Lactobacillus casei*) and nonprobiotic microflora in Argentinian Fresco cheese. J. Dairy Sci..

[35] Picot A., Lacroix C. (2004). Encapsulation of bifidobacteria in whey protein-based microcapsules and survival in simulated gastrointestinal conditions and in yoghurt. Int. Dairy J..

[36] RAPD-Primer Generator by J. Wöstemeyer, Institute of General Microbiology and Microbial Genetics, Germany. http://www2.uni-jena.de/biologie/mikrobio/tipps/rapd.html.

[37] Zhou M.Y., Celso E., Gomez-Sanchez C. (2000). Universal TA cloning. Curr. Issues Mol. Biol..

