# Supplementary material for: Molecular Detection of Two Potential Probiotic Lactobacilli Strains and Evaluation of Their Performance as Starter Adjuncts in Yogurt Production
Source: Int J Mol Sci. 2016 May 4;17(5):668. doi: 10.3390/ijms17050668 (PMC4881494; doi:10.3390/ijms17050668)
Supplement: Supplementary file 1 [file ijms-17-00668-s001.pdf]

Supplementary Materials: Molecular Detection of Two Potential Probiotic Lactobacilli Strains and Evaluation of Their Performance as Starter Adjuncts in Yogurt Production

Georgia Saxami, Olga S. Papadopoulou, Nikos Chorianopoulos, Yiannis Kourkoutas, Chrysoula C. Tassou and Alex Galanis

Table S1. The complete list of *Lactobacillus* strains tested in multiplex polymerase chain reaction (PCR) with the strain specific primer pair 281R/281F and the universal primer pair P1/P2. A unique 2-band pattern is only produced for *Lactobacillus pentosus* B281 (indicated as +). *Lactobacillus* strains that were negative in the current PCR assay with the strain specific primer pair 281R/281F are indicated as (−).

Stain Tested	PCR Specificity with Primer Pair	
	281F/281R	P1/P2
Lactobacillus pentosus B281	+	+
Lb. pentosus 141	−	+
Lb. pentosus E95	−	+
Lb. pentosus E106B	−	+
Lb. pentosus E128	−	+
Lb. pentosus E89	−	+
Lb. pentosus E119	−	+
Lb. pentosus E182	−	+
Lb. pentosus E105	−	+
Lb. pentosus 632	−	+
Lb. pentosus 612	−	+
Lb. pentosus 625A	−	+
Lb. pentosus E84	−	+
Lb. pentosus E120	−	+
Lb. pentosus 606	−	+
Lb. pentosus E83	−	+
Lb. pentosus 108	−	+
Lb. pentosus 139	−	+
Lb. pentosus E111	−	+
Lb. pentosus E110	−	+
Lb. pentosus E121	−	+
Lb. pentosus 97	−	+
Lb. pentosus E43	−	+
Lb. pentosus E101	−	+
Lb. pentosus E96	−	+
Lb. pentosus E129	−	+
Lb. pentosus 609	−	+
Lb. pentosus 637	−	+
Lb. plantarum B282	−	+
Lb. casei Shirota ACA-DC 6002	−	+
Lb. rhamnosus GG ATCC 53103	−	+
Lb. casei ATCC 393	−	+
Lb. zeae DSM 20178	−	+

Table S2. The complete list of *Lactobacillus* strains tested in multiplex PCR with the strains specific primer pair 282R/282F and the universal primer pair P1/P2. A unique 2-band pattern is only produced for *Lb. plantarum* B282 (indicated as +). *Lactobacillus* strains that were negative in the current PCR assay with the strain specific primer pair 281R/281F are indicated as (−).

Stain Tested	PCR Specificity with Primer Pair	
	282F/282R	P1/P2
Lactobacillus plantarum B282	+	+
Lb. plantarum E4	−	+
Lb. plantarum E1	−	+
Lb. plantarum E45	−	+
Lb. plantarum E50	−	+
Lb. plantarum E66	−	+
Lb. plantarum E68	−	+
Lb. plantarum E71	−	+
Lb. plantarum E73	−	+
Lb. plantarum E77	−	+
Lb. plantarum E79	−	+
Lb. plantarum 10	−	+
Lb. plantarum E69	−	+
Lb. plantarum E63	−	+
Lb. pentosus E43	−	+
Lb. pentosus B281	−	+
Lb. pentosus 390A	−	+
Lb. pentosus 97	−	+
Lb. pentosus E95	−	+
Lb. casei ATCC 393	−	+
Lb. paracasei DSM 20207	−	+
Lb. paracasei DSM 46331	−	+
Lb. paracasei DSM 5622	−	+
Lb. zeae DSM 20178	−	+
Lb. ingluviei DSM 15946	−	+
Lb. casei Shirota ACA-DC 6002	−	+
Lb. rhamnosus GG ATCC 53103	−	+
Lb. bulgaricus ATCC 11842	−	+
